# Are more GPs associated with a reduction in emergency hospital admissions? A quantitative study on GP referral in England

**DOI:** 10.3399/BJGP.2020.0737

**Published:** 2021-03-09

**Authors:** Catia Nicodemo, Barry McCormick, Raphael Wittenberg, FD Richard Hobbs

**Affiliations:** Head of Nuffield Department of Primary Care at Oxford University, Centre for Health Service Economics and Organisation, Nuffield Department of Primary Care Health Sciences, Radcliffe Observatory Quarter, Woodstock Road, and NIHR Oxford Biomedical Research Centre, Oxford.; Head of Nuffield Department of Primary Care at Oxford University, Centre for Health Service Economics and Organisation, Nuffield Department of Primary Care Health Sciences, Radcliffe Observatory Quarter, Woodstock Road, and NIHR Oxford Biomedical Research Centre, Oxford.; Head of Nuffield Department of Primary Care at Oxford University, Centre for Health Service Economics and Organisation, Nuffield Department of Primary Care Health Sciences, Radcliffe Observatory Quarter, Woodstock Road, and NIHR Oxford Biomedical Research Centre, Oxford.; Head of Nuffield Department of Primary Care at Oxford University, Centre for Health Service Economics and Organisation, Nuffield Department of Primary Care Health Sciences, Radcliffe Observatory Quarter, Woodstock Road, and NIHR Oxford Biomedical Research Centre, Oxford.

**Keywords:** deprivation, emergencies, emergency service, general practice, GP number, hospitalisation, instrumental variable, regression analysis

## Abstract

**Background:**

Recent studies have found an association between access to primary care and accident and emergency attendances, with better access associated with fewer attendances. Analyses of an association with emergency admissions, however, have produced conflicting findings.

**Aim:**

This study investigated whether emergency admission rates in an area are associated with 1) the number of GPs, and 2) mean size of GP practice.

**Design and setting:**

Analysis was conducted utilising Hospital Episode Statistics, the numbers of GPs and GP practices, Office for National Statistics population data, Quality and Outcomes Framework prevalence data, and Index of Multiple Deprivation data, from 2004/2005 to 2011/2012, for all practices in England.

**Method:**

Regression analysis of panel data with fixed effects to address 1) a potential two-way relationship between the numbers of GPs and emergency admissions, and 2) unobservable characteristics of GP practices.

**Results:**

There is not a statistically significant relationship between the number of GPs in a primary care trust area and the number of emergency admissions, when analysing all areas. In deprived areas, however, a higher number of GPs is associated with lower emergency admissions. There is also a lower emergency admission rate in areas in which practices are on average larger, holding GP supply constant.

**Conclusion:**

An increase in GPs was found to reduce emergency admissions in deprived areas, but not elsewhere. Areas in which GPs are concentrated into larger practices showed reduced levels of emergency admissions, all else being equal.

## INTRODUCTION

Recent Department of Health and Social Care (DHSC) policy goals include *‘a measurable reduction in age standardised emergency admission rates and emergency inpatient bed-day rates’* by 2020,^1^ while the DHSC 2016 Shared Delivery Plan comments that *‘Improved care in out-ofhospital settings is expected to lead to reduced need for emergency admissions to hospital’*.^2^

Emergency hospital admissions are urgent and unplanned admissions initiated by referral from accident and emergency (A&E), GPs, or ambulatory clinics, and have increased steadily in England from 4.4 million in 2004/2005 to 5.2 million in 2012/2013, an average annual rate of increase of 2.1%.^3^ Explanations for this growth include higher demand owing to:

- increases in illness;
- the increasing frailty of the ageing population;^4^ and
- changes in primary care, such as access to out-of-hours GP services.^5^^–^^6^

An emergency admission occurs when a patient is admitted to hospital urgently and unexpectedly (unplanned). Emergency admissions can occur via A&E, GPs, or consultants in ambulatory clinics.

GPs could influence the emergency admissions in several ways. Various recent UK studies have found, for example, that better access to primary care is associated with fewer A&E attendances.^7^^–^^10^ These studies suggest that a higher provision of GPs per 1000 population may be associated with fewer visits to emergency care, but do not analyse whether more GPs reduce the number of emergency admissions, with their implications for costs and patient care. Gulliford, in a study of 99 English health authorities,^11^ found that an increased supply of GPs was associated with lower hospital admissions for chronic and acute conditions. In a study of 68 practices, Harris and others^12^ found that access to primary health care does not explain differences between GP practices in potentially avoidable emergency attendances. In their study of 145 general practices, Bankart and others^13^ found that practice characteristics as well as various patient characteristics were associated with higher emergency admission rates. In a similar study in Northamptonshire, Gunther and others^14^ reached similar conclusions. In the US, it has been found that spending on health care in markets is lower in markets with a large proportion of primary care physicians.^15^^,^^16^

This paper investigates whether variations between areas in emergency admission rates are associated with variations of 1) the number of GPs per head, and 2) the average size of GP practices in an area, after allowing for various confounding factors. More GPs per head may enable practices to extend opening hours, diagnose illness before an emergency occurs, and offer patients rapid unplanned GP appointments. Clustering GPs into larger practices may attract patients away from emergency care by increasing timely access, and providing a wider range of specialist GP with extended roles services in the practice. However, areas with more (smaller) practices may be located closer to patients’ homes, and achieve greater continuity of care, both of which encourage primary care use.^17^

**Table:** How this fits in

Previous studies have produced mixed findings on the relationship between the number of GPs in an area and rate of emergency hospital admissions. This study presents new evidence on this issue using a large national panel dataset that links a range of data on primary and secondary care. It uses statistical techniques that take into account the potential two-way relationship between numbers of GPs and of emergency admissions. Deprived areas are associated with lower emergency admissions if the number of GPs increases.

Although these issues are straightforward, the existing literature is conflicting and has methodological shortcomings. Specific area-level healthcare policies may confound estimation of the relationship between GP supply and emergency admissions, as may any responsiveness of GP supply to evidence of high patient demand, which undermines treating GP supply as an exogenous explanatory variable. Two statistical techniques were used to address these two obstacles: a panel data approach to capture unobserved local-area differences with ‘fixed effects’, and an instrumental variables analysis to control for endogeneity. A unique dataset covering all registered patients in England for a relatively long time period (2004–2011) was used, which included data for population characteristics (index deprivation, expenditure per capita, etc.) and demand/need (Quality and Outcomes Framework [QOF]). Because the number of GPs was also controlled for, estimates of the effect of a larger mean size of practice on area admissions captured the implicit effect of having proportionately fewer such practices. Other datasets like Clinical Practice Research Datalink and QResearch cannot take into account where the practice is located or the characteristics of the practices. Another possible study design to estimate this type of problem is a structural equation model; however, as there are so many parameters and longitudinal data, attempting to estimate a structural equation model could make precisely estimating the parameters more difficult.

The changing supply of NHS GPs to areas of England from 2004–2011 was used to provide panel data estimates of their effect on emergency admissions, in a way which allows for the endogeneity of GP location choice. This longitudinal approach, which is more ’efficient’ than pooling cross-sections, helped to control for the impact of omitted variables and generates more accurate predictions for area outcomes.

## METHOD

### Data

A unique database was constructed for England linking several data sources at area level, for 2004–2011 (equivalent data for individual GPs are unavailable after 2012). The data were used in a longitudinal way, and all the variables needed to have values in each year selected: otherwise the models could not be estimated.

Hospital Episode Statistics (HES) on emergency hospital admissions were combined for the following:

- each Lower Super Output Area (LSOA);
- data on population by age, sex, and ethnicity at primary care trust (PCT) level produced by the Office for National Statistics (these variables were available for all the years selected only at PCT level);
- the Index of Multiple Deprivation (IMD) and the index of rurality at LSOA level;
- NHS Digital data on the number of full-time equivalent (FTE) GPs in each PCT;
- the number and the location of the practices;
- the number and the location of walk-in centres (WiC) and out-of-hours services (OOH);
- data on NHS expenditure per capita at PCT level; and
- QOF data on prevalence of specific diseases form each practice and aggregate at PCT level.

There were 151 PCTs and 32 482 LSOAs (average population 1500) in England in 2011. The variables and their sources are set out in Box 1. To measure the supply of GP services, HES data are linked with the ’General Medical Practices Exeter Payments’ data and the ’Practitioners of NHS Connecting for Health’ data. These databases contain information on current GPs and GP practices, and give both GP headcount and FTE information. The analysis and results for FTE GPs are presented in Results (see Supplementary Table S1 for the results for headcount GPs). Headcount is considered a robustness check on the main analysis.

**Box 1.** Description of variables and data sources

**Name of variable**	**Description**	**Data source**
**Emergency**	Total emergency admissions per 1K popn at LSOA level in the financial year	**HES**
**Female popn (%)**	Percentage of population female at LSOA level	**ONS**
**Female popn ≥60 (%)**	Percentage of female population aged ≥60 at LSOA level	**ONS**
**Male popn ≥65 (%)**	Percentage of male population aged ≥65 at LSOA level	**ONS**
**Black ethnicity (%)**	Percentage of black ethnicity at PCT level	**ONS**
**Asian ethnicity (%)**	Percentage of Asian ethnicity at PCT level	**ONS**
**Headcount GPs’ density at PCT 1K popn**	Number of GPs per 1K popn at PCT level	**NHS Digital**
**Ratio of practices at PCT 1K popn**	Number of GP practices at PCT per 1K popn	**NHS Digital**
**FTE GPs’ density at PCT 1K popn**	Number of FTE GPs per 1K popn at PCT level	**NHS Digital**
**Practice density at PCT 1K popn**	Number of GP practices per 1K popn at PCT level	**NHS Digital**
**WiC and OOH density at PCT 1K popn**	WIC and OOH centres per 1K popn at PCT level	**NHS Digital**
**Revenue per head**	HS expenditure per capita at PCT level	**DHSC**
**Deprivation areas**	Index of Multiple Deprivation at LSOAs in 10 deciles	**ONS**
**Prevalence of diseases**	Prevalence of specific diseases per 1K popn at PCT level from QOF	**NHS Digital**

DH = Department of Health and Social Care. FTE = full-time equivalent. HES = Hospital Episode Statistics.

HS = health expenditure. LSOA = Lower Super Output Area. ONS = Office for National Statistics. OOH = out-of-hours services. PCT = primary care trust. popn = population. QOF = Quality and Outcomes Framework. WiC = walk-in centres.

To take into account both traditional and new primary care delivery models, practice density at PCT level is measured using two variables: 1) the number of traditional GP practices per 1000 population (density of practices); and 2) the total number of WiCs and OOHs per 1000 population at PCT level. These variables are constructed at PCT level and not at LSOA level because many LSOAs have no practices, and this could bias the estimation. Undertaking the analysis at PCT level also allows control for the spatial correlation among LSOAs.

HES provides information on all patients admitted to NHS hospitals and NHS patients admitted to independent treatment centres in England. The total number of emergency admissions for each LSOA has been extracted.

QOF is a system used to remunerate GPs for providing good-quality care to their patients. The system, which covers almost all GP practices in England, includes prevalence rates for just 11 clinical conditions that are presented for all 7 years of this analysis: Coronary Heart Disease, Left Ventricular Dysfunction, Stroke and Transient Ischaemic Attack, Hypertension, Diabetes Mellitus, Chronic Obstructive Pulmonary Disease, Epilepsy, Hypothyroidism, Cancer, Mental Health, and Asthma. A QOF prevalence rate is the total number of patients on the register who has reported in the QOF data the health condition listed above, expressed as a proportion of the total number of patients registered with a practice at one point in time. The rate is calculated by grouping the prevalence of the 11 conditions at PCT level and dividing by the PCT population. To avoid endogenous recording of conditions following hospital admission, prevalence data for the year prior to the year of study for hospital admissions were used. The IMD combines information from seven domain indices to produce an overall relative measure of deprivation (the health domain has been excluded to avoid correlation with the other explicative variables), and gives each LSOA a rank from 1 (least deprived) to 10 (most deprived).

**Figure 1.**
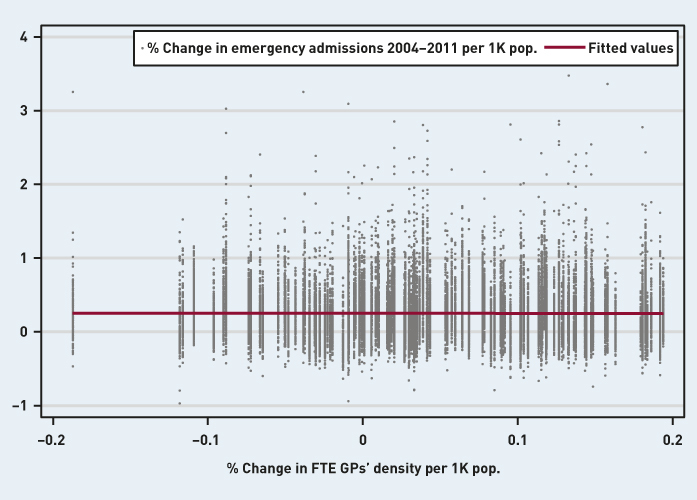
***Correlation between % change in FTE GPs’ density and emergency admissions.*** ***FTE = full-time equivalent. popn = population.***

The descriptive statistics for the variables used below are given in Table 1. Figure 1 illustrates the simple relationship in the data between the percentage change in GPs in each LSOA and changes in LSOA emergency admissions, between 2004–2011. This shows that there does not exist a simple relationship in which areas with greater growth in GPs per head have less growth in emergency admissions, prior to analysis of controls for confounding factors.

**Table 1.** Descriptive statistics at Lower Super Output Area (LSOA) level, 2004–2011

	**2004**	**2005**	**2006**	**2007**	**2008**	**2009**	**2010**	**2011**	**Total**
**Emergency admissions per 1K popn, % (SD)**	116.33 (44.20)	122.70 (45.66)	123.18 (46.92)	124.16 (48.17)	132.20 (49.41)	138.33 (51.59)	141.30 (52.73)	140.47 (52.04)	129.83 (49.73)
**Female popn, % (SD)**	51.04 (2.30)	51.01 (2.31)	50.98 (2.36)	50.95 (2.48)	50.92 (2.67)	50.90 (2.85)	50.87 (3.11)	50.97 (2.28)	50.96 (2.56)
**Female popn ≥60, % (SD)**	11.78 (4.59)	11.83 (4.61)	11.90 (4.65)	12.10 (4.73)	12.28 (4.82)	12.43 (4.88)	12.38 (4.82)	12.56 (4.94)	12.16 (4.77)
**Male popn ≥65, % (SD)**	6.87 (2.71)	6.92 (2.75)	6.96 (2.80)	7.02 (2.86)	7.14 (2.94)	7.28 (3.04)	7.42 (3.15)	7.27 (3.06)	7.11 (2.92)
**Black ethnicity, % (SD)**	2.60 (4.40)	2.67 (4.26)	2.72 (4.12)	2.78 (3.99)	2.84 (3.87)	2.88 (3.75)	2.93 (3.64)	2.91 (3.29)	2.79 (3.93)
**Asian ethnicity, % (SD)**	5.65 (6.89)	5.88 (6.80)	6.11 (6.71)	6.37 (6.66)	6.58 (6.57)	6.78 (6.50)	7.01 (6.46)	7.12 (6.08)	6.44 (6.61)
**PCT expenditure per head, % (SD)**	1.00 (0.69)	1.00 (0.69)	1.00 (0.68)	1.00 (0.68)	1.00 (0.13)	0.99 (0.13)	1.00 (0.14)	1.00 (0.14)	1.00 (0.49)
**Headcount GPs’ density at PCT per 1K popn, % (SD)**	0.83 (0.10)	0.87 (0.10)	0.89 (0.11)	0.93 (0.12)	0.96 (0.13)	1.00 (0.13)	1.04 (0.17)	1.06 (0.18)	0.95 (0.15)
**FTE GPs’ density at PCT per 1K popn, % (SD)**	0.61 (0.07)	0.62 (0.07)	0.65 (0.08)	0.64 (0.08)	0.66 (0.08)	0.68 (0.08)	0.68 (0.10)	0.67 (0.09)	0.65 (0.09)
**General practices’ density at PCT per 1K popn,^a^ % (SD)**	0.17 (0.04)	0.17 (0.04)	0.16 (0.04)	0.16 (0.04)	0.16 (0.04)	0.16 (0.04)	0.16 (0.04)	0.16 (0.04)	0.16 (0.04)
**WiC and OOH density at PCT per 1K popn,^b^ % (SD)**	0.00 (0.01)	0.01 (0.01)	0.01 (0.01)	0.01 (0.01)	0.01 (0.01)	0.01 (0.01)	0.01 (0.01)	0.01 (0.01)	0.01 (0.01)
**Observations, *N***	**32 482**	**32 482**	**32 482**	**32 482**	**32 482**	**32 482**	**32 482**	**32 482**	**259 856**

a Includes conventional partnership practices.

b Includes walk-in centres, out-of-hours centres, other prescribing cost centres, which include addiction services. FTE = full-time equivalent. LSOA = Lower Super Output Area. OOH = out-of-hours services. PCT = primary care trust. popn = population. SD = standard deviation. WiC = walk-in centres.

### Empirical strategy

Regression models are estimated at LSOA level to investigate how far rates of emergency hospital admission per 1000 population are associated with the number of GPs and the number of GP practices in the PCT, controlling for characteristics of the local population and for local disease prevalence rates. The model is as follows:

(1) $$F_{jt}=\beta X_{jt}+\alpha GP_{pt}+\rho GR_{pt}+Z_{jt}+d_{jt}+\omega_{jt}+\sigma_{j}+\mu_{t}+\varepsilon_{jt}$$

*F_jt_* represents the number of emergency admissions per 1000 population at each LSOA (*j*) in each year. The use of LSOA as the unit of observation has the advantage that it allows capture of the effects of variation of the explanatory variables across areas.

*X_jt_* is a vector of socioeconomic characteristics that are time varying at area *j* in time *t*. It includes the proportion of the PCT population in sex, age, and ethnicity groups and NHS expenditure per capita. *GP_pt_* is the number of FTE GPs per 1000 population in PCT *p* at time *t*. GR_pt_ is the number of GP practices per 1000 population at time *t* in PCT *p*. *Z_jt_* and *d_jt_* are dummy variables capturing respectively the urban/rural nature of the local area and local deprivation. *ω_jt_* is a vector of the local prevalence of the 11 QOF diseases. *μ_t_* and *σ_j_* are time and LSOA fixed effects respectively. *ε_jt_* is a random error term that is assumed to be normally distributed. Area fixed effect is an important factor here because time-invariant characteristics that are not observed at area level are controlled for.

Two versions of the variable for the number of GPs in an area were considered: headcount number of GPs and headcount number of FTE GPs. In this article, results are presented using the number of FTE GPs (for results using headcount see Supplementary Table S1).

As well as estimating the parameters of the model using ordinary least squares (OLS), the equation above is estimated using a two-stage least square (2SLS) or instrumental variables model. This technique is an extension of the OLS method to circumstances where the dependent variable’s error terms are correlated with an explanatory variable. In this study it is reasonable to be concerned that the measures of GP supply — the headcount number of GPs and the level of FTE GPs — may be partly determined by observed levels of the use of emergency care. For example, a greater number of FTE GPs could lead to an increase in the number of emergency admissions in an area because GPs may discover more cases given emergency admission. Also, if the emergency admissions increase in an area, the NHS could invest more resources in that area, attracting more GPs. In either case the number of GPs, and the hours each supplies, are not randomly assigned ‘doses’ across geographic areas as in a randomised clinical trial: unobservable factors could bias regression estimates. For these reasons OLS give biased estimates, and an instrumental variable approach is preferred.

An important criterion in choosing a good instrument to replace an endogenous regressor is that it is correlated with the endogenous regressor, but is only correlated with the dependent variable indirectly through its influence on the endogenous regressor. Two instruments are used; for FTE GPs the number of female GPs is used. This is the first time that this instrument has been used, to the best of the authors’ knowledge. The intuition behind this instrument is that female GPs are more likely to work part time in comparison with male GPs. The decision to work part time will affect the number of hours supplied, but the individual decision to work full time or part time is unlikely to be influenced by the level of emergency admissions in the area. As it is important to be sure that the effect of GPs on emergency admissions is captured, another variable is used to capture GP supply: the GP headcount. Again, this variable may be endogenous, and for that reason an instrumental variable is used. In this case the proportion of GPs who practiced in each area of England in 1980 is used. This approach was originally developed by Card in 2001.^18^ The main idea is to impute the number of GPs in an area based on the share of GPs in that area in the past to eliminate the recent, possibly endogenous movements of GP headcount (see Supplementary Appendix S1 for more detail on the instrumental-variable methods used).

## RESULTS

The findings are set out in Tables 2 and 3. The main explanatory variables are the number of FTE GPs and the number of places of primary care provision per 1000 population.

**Table 2.** The influence of the number of FTE GPs and of GP practices on emergency hospital admissions: OLS and 2SLS estimation^a^

	**OLS**						**2SLS**					

	**M1**			**M2**			**M3**			**M4**		

	Coef.	95% CI	*P*-value	Coef.	95% CI	*P*-value	Coef.	95% CI	*P*-value	Coef.	95% CI	*P*-value
**Female popn (%)**	0.465	0.334 to 0.593	<0.001	0.465	0.334 to 0.593	<0.001	0.467	0.327 to 0.583	<0.001	0.466	0.337 to 0.595	<0.001
% change	1.19			1.19			1.20	1.19				

**Female popn ≥60 (%)**	1.778	1.593 to 1.988	<0.001	1.770	1.5547 to 1.969	<0.001	1.782	1.587 to 1.979	<0.001	1.774	1.554 to 1.983	<0.001
% change	8.36			8.32			8.38			8.34		

**Male popn ≥65 (%)**	1.868	1.523 to 2.064	<0.001	1.858	1.525 to 2.064	<0.001	1.867	1.523 to 2.064	<0.001	1.854	1.515 to 2.055	<0.001
% change	5.42			5.39			5.41			5.38		

**Black ethnicity (%)**	1.378	1.024 to 1.836	<0.001	1.350	1.014 to 1.854	<0.001	1.392	1.045 to 1.912	<0.001	1.353	1.016 to 1.7566	<0.001
% change	5.37			5.27			5.43			5.28		

**Asian ethnicity (%)**	0.753	0.253 to 1.253	<0.01	0.66	0.143 to 0.520	0.08	0.80	0.354 to 1.276	<0.01	0.68	0.153 to 0.620	0.09
% change	4.97			na			5.28			na		

**Revenue per head**	−0.138	−1.073 to 0.519	0.494	−0.228	−1.073 to 0.519	0.398	−0.139	−0.986 to 0.715	0.423	−0.246	−1.068 to 0.515	0.786
% change	na			na			na			na		

**GPs’ density PCT 1K popn**	1.685	−18.136 to 35.388	0.188	−0.344	−12.672 to 64.289	0.127	−1.682	−13.573 to 57.256	0.681	−5.363	−8.842 to 2.837	0.314
% change	na			na			na			na		

**General practices density PCT 1K popn**	No			34.28	−11.672 to 44.378	0.563	No			45.535	0.365 to 76.589	<0.01
% change				na			na			1.37		

**WiC–OOH density PCT 1K popn**	No			Yes			No			Yes		

**Observations, *N***		253 376						253 376				

a Robust and cluster standard errors at PCT. Fixed effects are included for the year, LSOA, whether urban/rural, Index of Multiple Deprivation, and prevalence of disease. 2SLS = two stage least square. CI = confidence interval. coef. = coefficient. FTE = full-time equivalent. LSOA = Lower Super Output Area. na = not applicable. OLS = ordinary least squares.

OOH = out-of-hours services. PCT = primary care trust. popn = population. WiC = walk-in centres.

**Table 3.** The influence of the number of FTE GPs and of GP practices on emergency hospital admissions in deprived and relatively prosperous areas^a^

	**Deprived LSOAs**			**Most deprived LSOAs**			**Less deprived LSOAs**			**Least deprived LSOAs**		
			
	**IMD Ranks 8, 9, and 10**			**IMD Ranks 9 and 10**			**IMD Ranks 1, 2, and 3**			**IMD Ranks 1 and 2**		
			
	**2SLS**			**2SLS**			**2SLS**			**2SLS**		

	Coef.	95% CI	*P*-value	Coef.	95% CI	*P*-value	Coef.	95% CI	*P*-value	Coef.	95% CI	*P*-value
**Female popn (%)**	0.764	0.438 to 0.939	<0.001	0.805	0.480 to 1.131	<0.001	0.617	0.348 to 0.792	<0.001	0.613	0.333 to 0.894	<0.001
% change	2.03			2.16			1.65			1.65		

**Female popn +60 (%)**	2.107	1.762 to 2.561	<0.001	2.188	1.679 to 2.698	<0.001	1.576	1.234 to 1.815	<0.001	1.479	1.143 to 1.814	<0.001
% change	9.98			10.33			7.46			6.97		

**Male popn +65 (%)**	2.705	1.762 to 2.861	<0.001	2.889	2.249 to 3.528	<0.001	1.547	1.077 to 1.798	<0.001	1.406	0.939 to 1.874	<0.001
% change	7.97			8.12			4.54			4.06		

**Black ethnicity (%)**	1.962	1.049 to 2.407	<0.001	1.522	0.691 to 2.353	<0.001	0.056	−2.135 to 0.941	0.446	−0.286	2.422 to 1.850	0.793
% change	8.57			4.95			na			na		

**Asian ethnicity (%)**	1.039	1.595 to 2.180	0.08	1.906	1.087 to 2.725	<0.001	−1.885	−2.742 to −0.974	<0.001	−1.765	−2.913 to −0.617	<0.001
% change	na			12.58			−11.84			−11.22		

**PCT revenue per head**	−0.093	−1.368 to 0.959	0.73	0.349	−0.995 to 1.693	0.611	−0.761	−1.874 to 0.915	0.014	−1.096	−2.253 to 0.061	<0.01
% change	na			na			na			−0.436		

**GPs’ density PCT per 1K popn**	−12.812	−18.526 to −0.054	<0.01	−12.678	−23.529 to −1.827	<0.05	3.663	−4.003 to 9.832	0.409	−2.564	−10.880 to 5.753	0.546
% change	−10.76			−10.24			na			na		

**General practices’ density PCT per 1K popn**	68.401	−2.924 to 111.850	0.063	62.903	−3.949 to 129.755	<0.01	−8.965	−34.961 to 40.889	0.878	−13.25	−59.872 to 33.372	0.578
% change	25.99			24.34			na			na		

**Observations, *N***	89 762			50 160			89 762			50 160		

a Robust and cluster standard errors at PCT. Year, LSOA, urban/rural, deprivation, and prevalence disease fixed effects are included. 2SLS = two-stage least square. CI = confidence interval. coef. = coefficient. IMD = Index of Multiple Deprivation. FTE = full-time equivalent. LSOA = Lower Super Output Area. na = not applicable. PCT = primary care trust.

popn = population.

Estimates of four models are reported in Table 2: two OLS (M1 and M2) with and without control for practice size and treatment centres, and two 2SLS (M3 and M4) with and without equivalent controls. The analyses suggest that areas with more FTE GPs per head of population, all else being equal, do not have emergency admissions significantly different from areas with fewer GPs. This is consistent with Harris and others.^12^ The coefficient on the number of GPs when endogeneity is accounted for is larger and negative (M4), although not statistically significant. Increasing the number of GPs in an area is not found to influence the level of emergency admissions in the area.

A positive association is found between areas with a larger number of GP practices per head and those with a higher number of emergency admissions (holding constant the number of GPs), albeit only at a 10% significance level (M4). This implies that, holding constant the number of FTE GPs, there are more emergency admissions in areas with a larger number of smaller practices than in areas with a smaller number of larger practices: an increase in the mean number of practices of one standard deviation (SD) of the distribution of the number of GP practices across LSOAs will increase the number of emergency admissions by about 1.4%.

An increase in the proportion of the population who are female by one SD of the distribution of this proportion across LSOAs is associated with an increased rate of emergency admission of 1.2%. An increase in the proportion of the population who are females (males) aged ≥60 of the one SD higher distribution of this proportion across LSOAs increases the rates of emergency admission by 8.3% (5.3%). Similarly, an increase in the proportion of the population who are black by one SD of the variation across LSOAs increases the rate of emergency admissions by 5.3%. The proportion who are Asian have no significant influence on emergency admissions.

As there is evidence that both health state and use of health services vary with deprivation, it is considered that there are good a priori reasons for hypothesising that the relationship between number of GPs and the number of emergency admissions in an area might vary with deprivation. For example, even within disease categories, patient use of emergency care tends to be greater in less deprived areas, suggesting that primary care may work differently in deprived areas.^19^ Two samples of deprived areas are selected, using the IMD ranking of LSOA, based on a narrower and broader definition of deprived area. Similarly, two measures of relatively prosperous LSOAs are constructed. In Table 3 the estimation for the broad and narrow deprived LSOA area samples is presented in columns 1 and 2, and for the broad and narrow more prosperous LSOA area samples in columns 3 and 4.

This study finds that under both definitions of more prosperous areas (columns 3 and 4) neither the number of GPs nor the mean size of practice significantly affects the number of emergency admissions. However, in the two deprived area samples, the coefficient on the number of GPs is negative at 10% level of significance (column 1), and in the case of the smaller group of most deprived LSOAs the level of significance increases to 5% (column 2). This suggests that, under both definitions of deprived areas, a higher number of FTE GPs leads to a lower emergency admission rate: an increase in FTE GPs of one SD of the distribution of FTE per head of population across LSOAs will decrease the emergency admission rate by about 10% in both columns 1 and 2. The analysis also shows that, in these areas, patients of smaller practices have more emergency admissions on average than patients of larger practices: the number of emergency admissions is about 25% higher if the mean size of practice is decreased by one SD of the distribution of practice size across PCTs.

## DISCUSSION

### Summary

This study contains two main findings. First, an increase in the local level of FTE GPs per head of population, contrary to the common assumption, is not by itself associated with a reduction in emergency hospital admissions of patients resident in that local area, when other factors are held constant. In deprived areas, however, a higher number of GPs per head is associated with a lower emergency admission rate. Second, areas in which a given number of GPs are concentrated into fewer (larger by FTEs) practices have lower emergency admissions per head than otherwise similar areas with more (smaller) practices.

### Strengths and limitations

Panel data for all registered patients in England for 7 years from 2004/2005 were studied. Possible reverse causality (endogeneity) of GP location choice, variation in the prevalence of several diseases, and demographic variables were addressed. Although omitted variables owing to lack of data could be relevant, this is addressed by estimating fixed effects for each area and year. Separate analyses for admissions for different health conditions, which would have raised issues of small numbers at LSOA level, have not been conducted. The authors believe that the data (drawn from 2004/2005 to 2011/2012) and findings remain applicable to the situation now where the number of GPs has declined, and the population disease burden has increased. The analysis uses the number of GPs at PCT level, rather than at a local area level. As a consequence, it may not fully capture the associations between GP numbers and emergency admissions.

The study did not account for practice nurses and other non-GP clinical staff. Nurses and other staff do have a role in detecting some health conditions, as well as in chronic disease management, and larger practices may well employ more nurses. Neither was the performance of GPs considered: high quality of care would be expected to reduce the number of emergency admissions. Furthermore, although the prevalence rates of health conditions were controlled for using QOF data, as was the structure of the population (age, sex, deprivation, rurality and ethnicity) plus fixed effects at LSOA level, the QOF prevalence rates may be an underestimate of the rates of some conditions.

### Comparison with existing literature

This study’s findings are consistent with those of Harris and others,^12^ albeit the fact that they examined emergency department (self-referral, potentially avoidable) attendances in 2007/2008 and 2008/2009 rather than emergency admissions. They found that *‘avoidable emergency department attendance appears to be mostly driven by underlying deprivation rather than by the degree of access to primary care’*.^12^ The present findings differ, however, from those of Gulliford^11^ who examined the relationship between GP supply and indicators of population health in 1999. He found that each unit increase in GP supply was associated with a decrease in hospital admission rates for acute conditions and chronic conditions. This study used more recent data than Harris and others or Gulliford, and it also looked at all areas of England and all emergency admissions.

### Implications for practice

These findings suggest that increasing the number of FTE GPs in deprived areas could lead to fewer emergency hospital admissions of residents of these areas. Little connection was found in other areas between the number of FTE GPs and emergency admissions. The areas in which the available GPs became increasingly employed in large practices also appear to be areas with fewer emergency admissions. These findings may have helpful implications for policy towards fiscal incentives to encourage GPs to locate in deprived areas, and for addressing unmet demand for primary care.
